# Burns resulting from spontaneous combustion of electronic cigarettes: a case series

**DOI:** 10.1186/s41038-016-0061-9

**Published:** 2016-12-12

**Authors:** Clifford Sheckter, Arhana Chattopadhyay, John Paro, Yvonne Karanas

**Affiliations:** 1 Division of Plastic Surgery, Stanford University, Palo Alto, CA USA; 2 Santa Clara Valley Medical Center Regional Burn Center, 751 South Bascom Avenue, San Jose, CA 95128 USA

**Keywords:** Electronic cigarettes, E-cigarette, E-cig, Burn

## Abstract

**Background:**

Electronic cigarette (e-cigarette) sales have grown rapidly in recent years, coinciding with a public perception that they are a safer alternative to traditional cigarettes. However, there have been numerous media reports of fires associated with e-cigarette spontaneous combustion.

**Case Presentation:**

Three severe burns caused by spontaneous combustion of e-cigarettes within a 6-month period were treated at the Santa Clara Valley Medical Center Burn Unit. Patients sustained partial and full-thickness burns. Two required hospitalization and surgical treatment.

**Conclusions:**

E-cigarettes are dangerous devices and have the potential to cause significant burns. Consumers and the general public should be made aware of these life-threatening devices.

## Background

Electronic cigarettes (e-cigarettes) have become increasingly popular in the world and the USA as an alternative to traditional cigarettes. The public at large sees these products as a safer means of nicotine consumption and as a tool to aid smoking cessation [1]. Use of e-cigarettes has increased by over 200 % in the past 3 years in adults and increased ninefold in adolescents [2–4]. There are currently no scientific data that demonstrate that electronic cigarettes are any safer than traditional cigarettes. While public perceptions on safety primarily relate to historical health problems with tobacco products such as lung cancer and chronic pulmonary disease, there are no reports regarding how the public actually perceives the safety of the e-cigarette devices. However, there have been documented events in the media of spontaneous combustion events involving the electronic cigarette device [5–7]. Further, there have been case reports in both the USA and UK of e-cigarettes causing thermal, blast, and alkali burns from spontaneous combustion [8–10]. Most of these burns were sustained to the thigh as e-cigarettes combusted in a pant pocket, although two patients sustained facial trauma and burns while smoking. The maximum size of these reported burns was 4 % total body surface area (TBSA).

Our institution has encountered the immediate health effects of such combustion events. Two of three instances resulted in full-thickness burns requiring inpatient admission and surgery for excision and skin grafting. Herein, we report the details of these cases and raise awareness for the danger of electronic cigarette use.

We performed a retrospective chart review of all patients admitted to the Santa Clara Valley Medical Center Burn Center from July 2015 to April 2016. Patients who were treated for e-cigarette-related burns were identified. Their cases were reviewed to determine surgical treatments, extent of burns, depth of burns, length of stay, complications, and outcomes. Institutional Review Board approval and patient consent were obtained.

## Case presentation

Three cases were identified. The average burn TBSA was 8 %, ranging from 2 to 15 %. All patients had a mixture of partial- and full-thickness burns. The average length of stay was 9 days ranging from 1 to 16 days. Two patients required inpatient admission and surgery. All the patients were male and habitual e-cigarette users. The average age at time of injury was 29 years old.

### Case 1

A 34-year-old male with a history of ulcerative colitis and opioid dependence had placed an electronic cigarette in his right pant pocket which spontaneously combusted while shopping in public. His pants caught fire and were immediately removed to extinguish flames. He was brought to the emergency department by an ambulance.

**Fig. 1:**
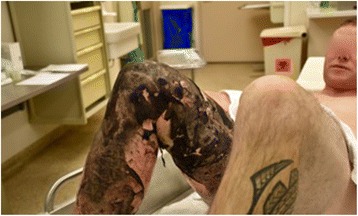
Case 1, the right leg on admission

**Fig. 2:**
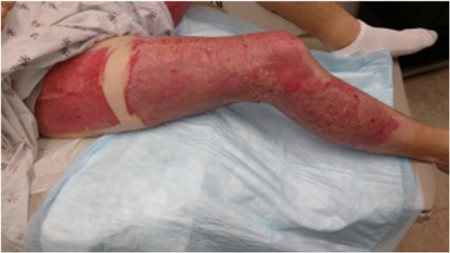
Case 1, postoperative lateral view of the right leg

**Fig. 3:**
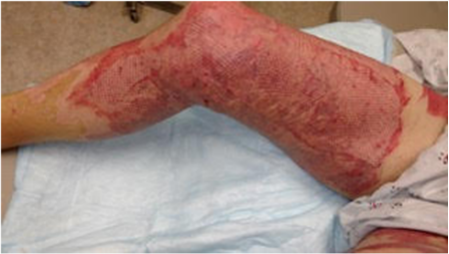
Case 1, postoperative medial view of the right leg

Examination revealed a 15 % TBSA circumferential deep partial-thickness and full-thickness burn of the right leg (Fig. 1). An epidural was placed for pain control, and on post-burn day 2, the patient was taken to the operating room for tangential excision with skin allograft placement to a 2000 cm^2^ surface area. The allografted areas were dressed with N-Terface (MEDLINE Industries, Inc., IL, USA) and ACTICOAT (Smith & Nephew, London, UK), and the debrided superficial partial-thickness burns were dressed with Mepilex Ag (Molnlycke, Gothenburg, Sweden). On post-burn day 6, the allograft was removed, revealing healthy subcutaneous tissue ready for skin autografting. Two thousand four hundred square centimeters of meshed split-thickness skin grafts were used to cover the wounds. The autografts showed an excellent take on post-burn day 9, and the patient was discharged home on post-burn day 16 (Figs. 2 and 3).

### Case 2

A 19-year-old otherwise healthy male had an electronic cigarette in his left pant pocket when it combusted and lit his pants on fire. He immediately removed his pants and rolled on the ground, which extinguished the flames. He was brought to the emergency department by an ambulance.

**Fig. 4:**
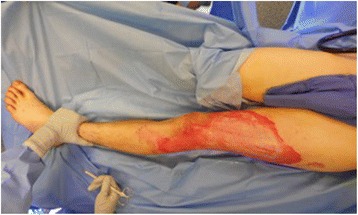
Case 2, preoperative lateral view of the left leg

**Fig. 5:**
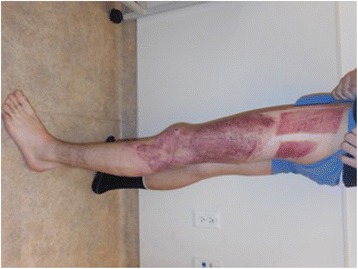
Case 2, postoperative lateral view of the left leg

Examination revealed a 7 % TBSA non-circumferential mixed partial- and full-thickness burn to the lateral thigh and calf (Fig. 4). On post-burn day 3, he was taken to the operating room for tangential excision and immediate split-thickness skin autografting of 800 cm^2^. The patient was discharged on post-burn day 9 with 100 % graft take and healing wounds (Fig. 5).

### Case 3

**Fig. 6:**
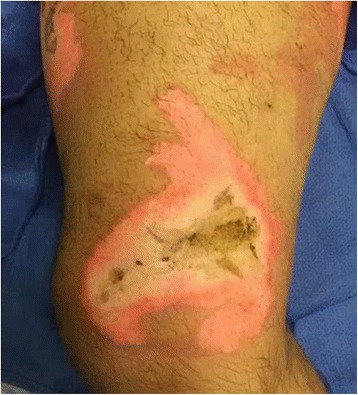
Case 3, medial right thigh above the knee

**Fig. 7:**
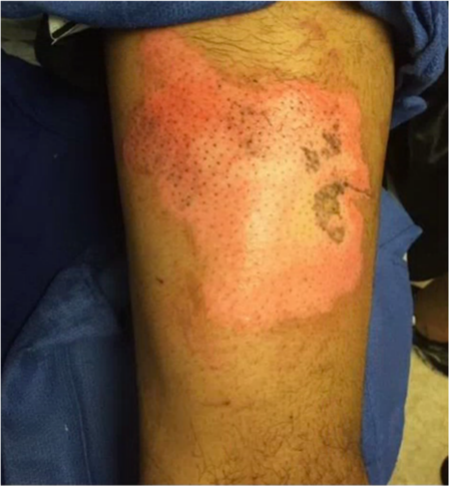
Case 3, anterior right thigh

A 35-year-old otherwise healthy male sustained a 2 % TBSA burn to his right lateral thigh when an e-cigarette device in the right back pocket of his pants spontaneously combusted, burning a hole through his pants (Fig. 6). Upon arrival to the emergency department, he was found to have partial- and full-thickness burns to his right thigh (Fig. 7). The patient was treated in the outpatient setting with topical wound care. The patient elected for non-operative treatment and healed in 5 weeks.

## Conclusions

Electronic cigarettes have been associated with at least 25 media reports of fires since 2009. This is likely an underestimation of the problem, as our institution encountered three e-cigarette burn victims in the span of 6 months. Furthermore, we illustrate two patients with 7 and 15 % TBSA burns, larger than the previously reported e-cigarette burns [8–10]. Eighty percent of these reported fires were associated with charging e-cigarettes in built-in USB ports in cars [11]. However, the voltage and current delivered to USB ports vary greatly, leading to “overcharging", excess thermal energy, and subsequent explosions.

**Fig. 8:**
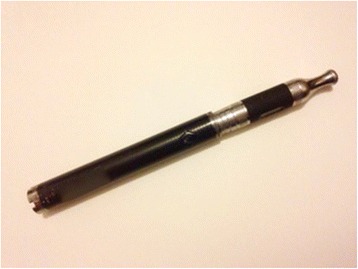
Common electronic cigarette device containing a lithium-ion battery

All the patients in this report experienced e-cigarette combustion while carrying the e-cigarette device with a battery in a pocket. The cause of this type of explosion is a suspected failure of the lithium-ion battery interface. E-cigarettes contain a cylindrical battery enclosed in a thin metal cylinder with structural weakness at the ends (Fig. 8). When the electrolyte within the battery overheats, the cylinder is unable to withstand the internal pressure that builds up and ruptures the battery, causing an explosion. The thermal energy from the explosion is clearly able to cause full-thickness burns leading to permanent disfigurement with risk for loss of limbs and life. In some cases, inpatient treatment and multiple surgeries are needed for effective treatment. Not only that this severely limits the lives of those affected but there is also a greater societal cost to the health system in managing these injuries.

In addition to the multiple ways that e-cigarettes cause fires, they also present a broader public health risk. A longitudinal cohort study found that e-cigarette users aged 16–26 were over eight times more likely to progress to traditional cigarettes compared to non-users [12]. E-cigarettes act as a “nicotine starter,” slowly addicting users with fewer adverse side effects than traditional cigarettes. Eventually, as e-cigarette users become tolerant, they seek out the higher nicotine doses found in traditional cigarettes. Thus, the idea that e-cigarettes are a safer way to smoke is now becoming defunct.

Electronic cigarette devices spontaneously combust, have the ability to cause full-thickness burns, and act as a gateway to traditional cigarette use. While the public views these devices as benign and believes they offer a safer means to consume tobacco, these devices are dangerous and pose a serious public health risk.

## References

[1] Nonnemaker J, Kim AE, Lee YO, MacMonegle A. Quantifying how smokers value attributes of electronic cigarettes. Tob Control. 2016;25(e1):e37-43. 10.1136/tobaccocontrol-2015-052511 26546152

[2] Barrington-Trimis JL, Berhane K, Unger JB, Cruz TB, Huh J, Leventhal AM, Urman R, Wang K, Howland S, Gilreath TD, Chou C-P, Pentz MA, McConnell R (2015). Psychosocial factors associated with adolescent electronic cigarette and cigarette use. Pediatrics.

[3] Dutra LM, Glantz SA (2014). Electronic cigarettes and conventional cigarette use among U.S. adolescents: a cross-sectional study. JAMA Pediatr.

[4] Wasowicz A, Feleszko W, Goniewicz ML (2015). E-Cigarette use among children and young people: the need for regulation. Expert Rev Respir Med.

[5] Kulwicki MF. Child burned after e-cigarette explodes in car charger. Fox [Internet]. Salt Lake City; 2013. Available from: http://fox13now.com/2013/09/21/child-burned-after-e-cigarette-explodes-in-car-charger/?hpt=us_bn10. Accessed 25 May 2016.

[6] Branson-Potts H. Woman burned by exploding e-cigarette battery awarded $1.9 million. Los Angeles Times [Internet]. 2015. Available from: http://www.latimes.com/local/crime/la-me-ecigarette-burns-verdict-20151001-story.html. Accessed 25 May 2016.

[7] Rogér JM, Abayon M, Elad S, Kolokythas A (2016). Oral trauma and tooth avulsion following explosion of e-cigarette. J Oral Maxillofac Surg.

[8] Kumetz EA, Hurst ND, Cudnik RJ, Rudinsky SL. Electronic cigarette explosion injuries: a case series. The American Journal of Emergency Medicine. 2016. 10.1016/j.ajem.2016.04.010 27133537

[9] Nicoll KJ, Rose AM, Khan MAA, Quaba O, Lowrie AG (2016). Thigh burns from exploding e-cigarette lithium ion batteries: first case series. Burns.

[10] Colaianni CA, Tapias LF, Cauley R, Sheridan R. Injuries caused by explosion of electronic cigarette devices. Eplasty. 2016;16:ic9. PMC4778159 26966477

[11] United States Fire Administration. Electronic cigarette fires and explosions. FEMA [Internet]. 2014. Available from: https://books.google.com/books?id=dqoYrgEACAAJ. Accessed 25 May 2016.

[12] Primack BA, Soneji S, Stoolmiller M, Fine MJ, Sargent JD (2015). Progression to traditional cigarette smoking after electronic cigarette use among US adolescents and young adults. JAMA Pediatr.

